# Impact of acute respiratory distress syndrome on outcome in critically ill patients with liver cirrhosis

**DOI:** 10.1038/s41598-025-88606-z

**Published:** 2025-02-04

**Authors:** Pischtaz Adel Tariparast, Kevin Roedl, Thomas Horvatits, Andreas Drolz, Stefan Kluge, Valentin Fuhrmann

**Affiliations:** 1 Department of Intensive Care Medicine, University Medical Center Hamburg-Eppendorf, Martinistr. 52, 20246 Hamburg, Germany; 2 Department of Medicine I, University Medical Center Hamburg-Eppendorf, Hamburg, Germany; 3 Department of Medicine and Gastroenterology, Heilig Geist-Hospital, Cologne, Germany

**Keywords:** Liver cirrhosis, Acute-on-chronic liver failure, Multiple organ failure, Intensive care unit, Acute liver failure, ARDS, Respiratory distress syndrome, Outcomes research

## Abstract

We investigated the occurrence and outcome of respiratory failure and ARDS in critically ill patients with liver cirrhosis. This is a retrospective analysis of patients with liver cirrhosis at an ICU during an 8-Year period. An assessment of acute on chronic liver failure as well as the presence and grade of ARDS within the first 72 h of admission to the ICU was performed. A total of 735 patients during the study period. Median age was 58 (50–69) years and 61% (*n* = 447) were male. 57% (*n* = 421) of the patients received mechanical ventilation (MV). Liver specific as well as ICU scores on admission were significantly higher in patients with MV. Necessity of vasopressor support (86%vs.25%, *p* < 0.001) and RRT (50%vs.11%, *p* < 0.001) was more frequent in patients with MV. The incidence of ARDS within the first 72 h of admission was 8% (*n* = 61). We observed a 28-day mortality or liver transplantation rate of 54% (*n* = 196) and 66% (*n* = 66%) in patients with MV and ARDS, respectively. After 90-days 63% (*n* = 226) with MV and 70% (*n* = 43) with ARDS were dead or received liver transplantation. ARDS is a prognostic factor for mortality in patients with liver cirrhosis admitted to the ICU. One out of ten critically ill cirrhotic patients develop ARDS within 72 h after admission. Although mortality rates are high initially critical care therapy should not be withheld and must be reevaluated regularly.

**Supplementary Information:**

The online version contains supplementary material available at 10.1038/s41598-025-88606-z.

## Introduction

Respiratory failure is a less frequent type of organ failure in patients with liver cirrhosis and acute-on-chronic liver failure (ACLF) admitted to the intensive care unit (ICU)^1^. The respiratory function in patients with liver cirrhosis can be affected by pulmonary vascular changes presenting as hepatopulmonary syndrome with intrapulmonary shunting^2–4^ or extrapulmonary changes like ascites or hepatic hydrothorax^5^. Furthermore, also common causes of respiratory failure like pneumonia, pulmonary oedema and pleural effusion can cause respiratory deterioration in patients with liver cirrhosis.

Admission to ICU of patients with liver cirrhosis is associated with increased mortality and worse outcome^6–8^. Further, need for mechanical ventilation (MV) was associated with mortality in patients with liver cirrhosis as shown previously^6,9–11^. However, there is only very limited data concerning presence of ARDS in patients with liver cirrhosis^12^. Furthermore, it is currently unknow how patient outcome is affected regarding presence of different ARDS stages (mild, moderate or severe). Although ARDS stage is associated with mortality its clinical relevance is unclear in patients with liver cirrhosis. Clinical data could help in early decision making in critically ill patients with ACLF and simultaneous severe respiratory failure.

Therefore, the main objective of this study was to investigate the occurrence and outcome of early respiratory failure in critically ill patients with liver cirrhosis. Further, it should be investigated if presence of ARDS in patients with liver cirrhosis is associated with adverse outcome and can be used for early risk stratification.

## Patients and methods

We conducted a retrospective analysis based on prospectively collected data. We included all patients with proven liver cirrhosis admitted to the department of intensive care medicine at the University Medical Centre Hamburg-Eppendorf (Germany) from 01/2009 to 12/2016. Cause of ICU admission and comorbidities were assessed and documented. Admission grade of acute-on-chronic liver failure (ACLF) was registered. Presence of Acute respiratory distress syndrome (ARDS) was defined according to the Berlin definition^13^ and assessed in the first 72 h of ICU admission. Further, vasopressor therapy and dosage, need for renal replacement therapy (RRT), need for mechanical ventilation and routine laboratory was recorded in all patients. Patients that previously underwent liver transplantation were excluded from analysis.

Severity of illness was evaluated by sequential organ failure assessment (SOFA)^14^, simplified acute physiology (SAPS II)^15^ score, Model of End-stage Liver Disease (MELD) score^16^, Chronic Liver Failure-Sequential Organ Failure Assessment (CLIF–SOFA) score^1^, Chronic Liver Failure Consortium acute-on-chronic liver failure (CLIF-C ACLFs) score^1,17^ and lactate-adjusted CLIF-C ACLFs (CLIF-C ACLFs _Lactate_)^18^ score were calculated as previously described on ICU admission.

Furthermore Charlson Comorbidity Index (CCI)^19^ was calculated in all patients. Hepatic encephalopathy (HE) was graded according to West-Haven criteria^20^. Sepsis and septic shock were diagnosed according to well established criteria^21,22^.

Intensive and critical care management of all patients was performed following local standard of care procedures that were derived from accepted recommendations and guidelines for the treatment of critically ill cirrhotic patients^23–26^. Intravenous fluid administration as well as vasopressor therapy was initiated in patients meeting shock criteria aiming to maintain a mean arterial blood pressure of > 65 mmHg. Broad-spectrum antibiotic therapy was initiated in patients with suspected or proven bacterial infection according to standardized protocols; antimicrobial therapy was adapted to culture results. Renal replacement therapy (RRT) was initiated in patients with renal failure and/or metabolic acidosis^27^. Patients with cirrhosis with signs of HE received lactulose. Patient`s showing HE ≥ grade 3 were intubated and mechanically ventilated. The mechanical ventilation strategy followed the lates guidelines including a protective ventilation strategy^28,13^.

Twenty-eight-day-mortality, 90-day-mortality, and 1-year mortality was assessed on site or by contacting the patients or their attending physicians. Data were documented prospectively in the patient data management system by trained staff. The Ethics Committee of the Hamburg Chamber of Physicians (“Ethikkomission der Ärztekammer Hamburg”) approved the study (No.: WF-014/19). Owing to the retrospective character of the study and its pseudonymised data collection, the need for informed consent was waived by the Ethics Committee of the Hamburg Chamber of Physicians (“Ethikkomission der Ärztekammer Hamburg”). The study was conducted in compliance with the Declaration of Helsinki as well as in accordance with local guidelines and regulations.

### Definitions

#### Liver cirrhosis

Liver cirrhosis was diagnosed based on a combination of clinical characteristics (e.g., ascites, caput medusae, spider angiomata, etc.), laboratory and radiological findings (typical morphological changes of the liver, sings of portal hypertension, etc. in abdominal ultrasonography or computed tomography scan), or via histology, if available^29,30^.

#### Acute-on-chronic liver failure

ACLF was diagnosed and graded according the well-established definition of the CLIF consortium^1^, as follows: Liver Failure was defined as serum bilirubin ≥ 12 mg/dl; Renal failure: serum creatinine ≥ 2 mg/dl; Cerebral failure: grade III-IV hepatic encephalopathy (West-Haven classification); Coagulation failure: international normalized ratio (INR) ≥ 2.5; Circulatory failure: use of vasoconstrictors to treat severe arterial hypotension (use of vasoconstrictors for the treatment of HRS in patients without severe hypotension not included); Respiratory failure: PaO2/FiO2 ≤ 200 or SpO2/FiO2 ≤ 214. Renal dysfunction was diagnosed when serum creatinine ranged between 1.5 and 1.9 mg/dl; cerebral dysfunction was diagnosed in patients with Grade I or Grade II hepatic encephalopathy. ACLF grade 1 includes patients with renal failure alone or of any other type of single organ failure if associated to renal dysfunction and/or cerebral dysfunction. ACLF grade 2 includes patients with 2 organ failures, whereas ACLF grade 3 includes patient`s ≥ 3 organ failures.

#### ARDS

ARDS was defined according to the Berlin definition, using the PaO2/FiO2 ratio (Horowitz index) as marker for severity^13^. Clinical patient management od ARDS was performed according to national and international guidelines, including prone positioning in moderate to severe ARDS, restrictive fluid management following the initial resuscitation period. Patients with severe hypoxemic and/or hypercapnic respiratory failure in combination with severe respiratory acidosis refractory to adjunctive therapies were evaluated for vv-ECMO therapy. Criteria for the initiation of vv-ECMO support were based on the guidelines of the Extracorporeal Life Support Organization (ELSO) and national recommendations^31–33^.

### Statistics

Data are presented as count and relative frequency or median and 25–75% interquartile range (IQR). Clinical variables were compared by Chi-squared, Fisher exact or Mann-Whitney U test for hypothesis testing as appropriate. Correlation analysis was performed using Spearmen`s correlation. Multivariable Cox proportional hazard analysis was performed to assess predictors of mortality. A forward stepwise procedure was used to identify most potent predictors. Survival function estimates were calculated using the Kaplan-Meier Method and were compared by the log-rank test. Statistical analysis was conducted using IBM SPSS Statistics Version 24.0 (IBM Corp., Armonk, NY). Generally, a p-value < 0.05 was considered statistically significant.

## Results

### Study population and baseline characteristics

**Fig. 1:**
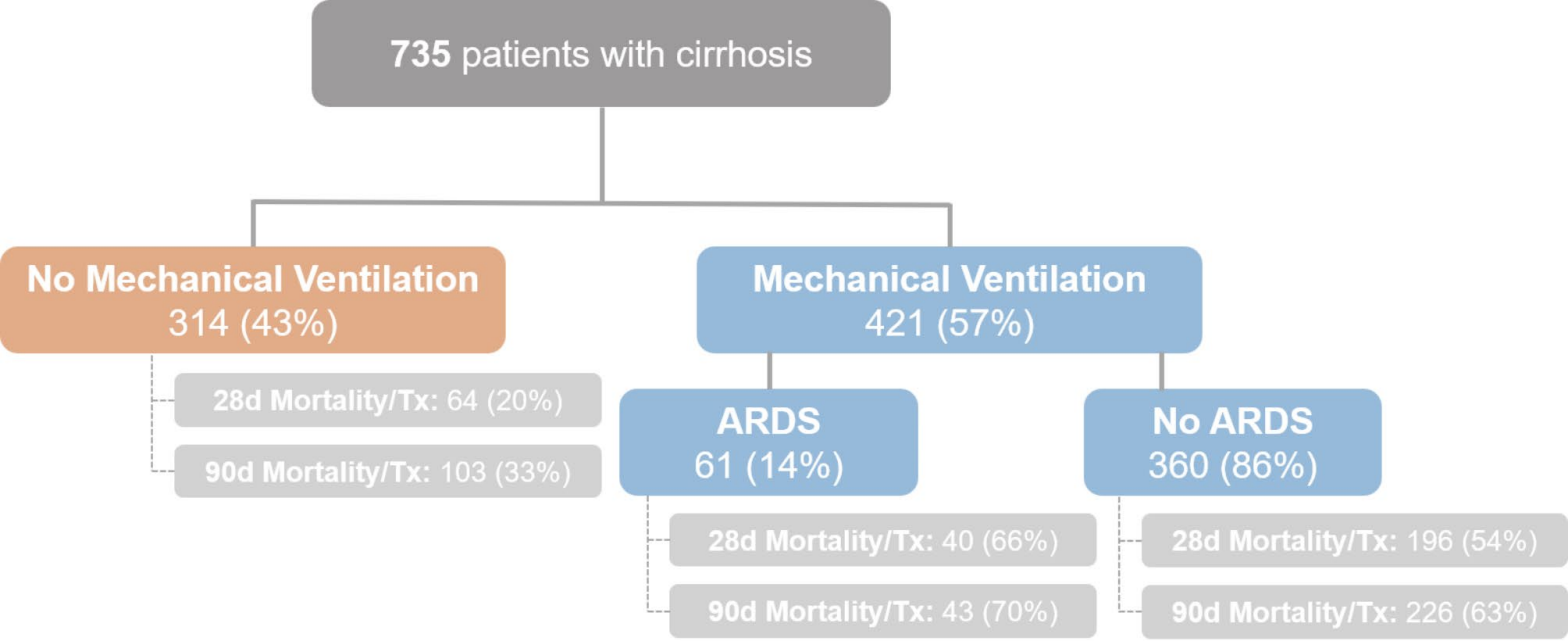
Flow-Chart of the study.

735 critically ill patients with liver cirrhosis were included in the study. Of those, 57% (*n* = 421) received mechanical ventilation (MV) and 43% (*n* = 314) did not (see Study Flow-Chart Fig. 1). The median age of the included patients was 58 (50–66) years and 61% (*n* = 447) were male. The main underlying causes of liver cirrhosis was alcoholic liver disease in 64% (*n* = 468). The median MELD and Child-Pugh score on admission were 21 (13–30) and 10 (7–11) points, respectively. The median SOFA score was 10 (6–14) points and SAPS II on admission was 40 (28–56) points. CLIF-SOFA and CLIF-C ACLF score were 12 (6–15) and 54 (47–59) points on admission. 60% (*n* = 440) had vasopressor support, 57% (*n* = 421) received MV and 33% (*n* = 245) required renal replacement therapy (RRT). The median length of hospital and ICU stay was 17 (9–30) and 4 (1–9) days, respectively. In the whole cohort 28-day and 90-day mortality or liver transplantation was 41% (*n* = 300) and 51% (*n* = 372), respectively. Detailed baseline characteristics of the cohort are shown in Table 1.

**Table 1:** Baseline characteristics of the study population.

Variables	All patients (*n* = 735)
Age, years (*median; IQR)*	58 (50–66)
Sex, male (*n*,* %)*	447 (61)
Etiology of liver cirrhosis, (*n*,* %)* - Alcoholic	468 (64)
Therapy - ICU *(n*,* %)*	
- Mechanical ventilation	421 (57)
- Vasopressor therapy	440 (60)
- Renal Replacement therapy	245 (33)
Scores, points (*median; IQR)*	
- MELD	20 (13–28)
- Child-Pugh	10 (7–12)
- SOFA	10 (6–14)
- SAPS II	40 (28–56)
- CLIF-SOFA	12 (6–15)
- CLIF-C ACLF	54 (47–59)
- CLIF-C ACLF _Lactate_	54 (45–61)
Outcome, (*n*,* %)*	
- LOS – ICU, days (*median; IQR)*	4 (1–9)
- LOS – Hospital, days (*median; IQR)*	17 (9–30)
- 28-day Mortality/tx (*n*,* %)*	300 (41)
- 90-day Mortality/tx (*n*,* %)*	372 (51)

*Abbreviations*: IQR, interquartile range; n, number; ICU, intensive care unit; MELD, Model of End Stage Liver Disease; SOFA, sequential organ failure assessment; SAPS, simplified acute physiology score; CLIF, Chronic Liver Failure; ACLF, Acute-on-chronic liver failure; LOS, length of stay; tx, transplantation;

### Clinical characteristics of patients with mechanical ventilation compared to patients without mechanical ventilation

Detailed characteristics of patients with and without MV are shown in Table 2. Median age and sex were comparable in both groups. Cause of ICU admission was gastrointestinal bleeding in 25% (*n* = 105) and 17% (*n* = 52), infection/sepsis in 18% (*n* = 76) and 10% (*n* = 30), surgical in 19% (*n* = 82) and 28% (*n* = 88), hepatic encephalopathy in 9% (*n* = 36) and 8% (*n* = 26), renal failure in 6% (*n* = 25) and 9% (*n* = 28) in patients with and without MV, respectively. 96% (*n* = 402) of patients with MV and 47% (*n* = 149) presented with ACLF on admission. Liver specific as well as ICU scores on admission were significantly higher in patients with MV. Necessity of vasopressor support (86% vs. 25%, *p* < 0.001) and RRT (50% vs. 11%, *p* < 0.001) was more frequent in patients with MV. Blood gas analysis on admission revealed significant differences according paO_2_ (98 vs. 90 mmHg, *p* = 0.001), paCO_2_ (38 vs. 35 mmHg, *p* = 0.034), pH-level (7.33 vs. 7.39, *p* < 0.001) and lactate (2.7 vs. 1.7 mmol/l, *p* < 0.001). We observed a significantly higher 28-day and 90-day mortality or liver transplantation rate in patients with MV (all timepoints *p* < 0.001) (see Table 2; Fig. 1). The 28-day and 90-day mortality or liver transplantation rate was 56% vs. 20%, 63% vs. 33% in patients with and without MV, respectively (See Fig. 1; Table 2).

**Table 2:** Characteristics of the study population stratified according mechanical ventilation.

Variables	MV (*n* = 421)	No-MV (*n* = 314)	*p* value
Age, years (*median; IQR)*	58 (51–65)	58 (49–66)	0.49
Sex, male (*n*,* %)*	265 (63)	182 (58)	0.19
Cause of ICU admission, (*n*,* %)*			< 0.001
- Infection/Sepsis	76 (18)	30 (10)	
- Bleeding	105 (25)	52 (17)	
- Renal failure/Hepatorenal Syndrome	25 (6)	28 (9)	
- Cardiovascular	3 (1)	7 (2)	
- Hepatic encephalopathy	36 (9)	26 (8)	
- Surgery/Intervention	82 (19)	88 (28)	
- Other	94 (22)	83 (26)	
MELD score, points (*median; IQR)*	22 (14–30)	16 (12–24)	< 0.001
Child-Pugh score, points (*median; IQR)*	10 (8–12)	9 (7–11)	< 0.001
SOFA, points (*median; IQR)*	13 (11–16)	5 (3–8)	< 0.001
SAPS II, points (*median; IQR)*	52 (38–65)	29 (23–38)	< 0.001
CLIF-SOFA, points (*median; IQR)*	14 (12–17)	6 (4–10)	< 0.001
CLIF-C ACLF, points (*median; IQR)*	57 (52–61)	48 (43–54)	< 0.001
CLIF-C ACLF _Lactate_, points (*median; IQR)*	57 (51–66)	46 (39–54)	< 0.001
Therapy, (*n*,* %)*			
- Vasopressor support	361 (86)	79 (25)	< 0.001
- Mechanical Ventilation	421 (100)	0 (0)	-
- Renal Replacement therapy	212 (50)	33 (11)	< 0.001
ACLF grade - on admission*, (*n*,* %)*			< 0.001
- No ACLF	16 (4)	158 (50)	
- ACLF grade 1	25 (6)	64 (20)	
- ACLF grade 2	66 (16)	54 (17)	
- ACLF grade 3	311 (74)	31 (10)	
Vital signs – on admission, (*median; IQR)*			
- Heart rate, bpm	87 (72–102)	88 (74–101)	0.67
- Mean arterial pressure, mmHg	74 (64–86)	81 (70–93)	< 0.001
- Body temperature, C°	35.7 (34.7–36.6)	36.4 (35.8–36.8)	< 0.001
Blood gas analysis – on admission, (*median; IQR)*			
- paO_2_, mmHg	98 (77–138)	90 (75–110)	0.001
- paCO_2_, mmHg	38 (30–44)	35 (30–42)	0.034
- pH, level	7.33 (7.25–7.41)	7.39 (7.34–7.44)	< 0.001
- Base-Excess, mmol/l	-5 (-11–1)	-3 (-8–1)	< 0.001
- Bicarbonate, mmol/l	20 (16–24)	22 (19–25)	< 0.001
- Lactate, mmol/l	2.7 (1.5–6.2)	1.7 (1.1–2.7)	< 0.001
Laboratory – on admission, (*median; IQR)*			
- Leukocytes, G/l	9.9 (6.5–15.8)	8.5 (5.9–12.6)	0.001
- Creatinine, mg/dl	1.6 (1.0–2.7)	1.3 (0.8–2.3)	< 0.001
- Bilirubin, mg/dl	2.5 (1.2–7.4)	2.1 (1.1–5.2)	< 0.001
- CRP, mg/dl	14 (10–58)	17 (6–55)	0.007
- INR	1.5 (1.2–1.9)	1.4 (1.2–1.6)	< 0.001
Outcome, (*n*,* %)*			
- LOS – ICU, days (*median; IQR)*	7 (3–15)	2 (1–4)	< 0.001
- LOS – Hospital, days (*median; IQR)*	18 (9–34)	16 (9–28)	0.38
- 28-day Mortality/tx (*n*,* %)*	236 (56)	64 (20)	< 0.001
- 90-day Mortality/tx (*n*,* %)*	266 (63)	103 (33)	< 0.001

*Abbreviations*: IQR, interquartile range; n, number; MV, mechanical ventilation; ICU, intensive care unit; MELD, Model of End Stage Liver Disease; SOFA, sequential organ failure assessment; SAPS, simplified acute physiology score; CLIF, Chronic Liver Failure; ACLF, Acute-on-chronic liver failure; CRP, C Reactive Protein; INR, international normalized ratio; LOS, length of stay; tx, transplantation;

* 10 missing;

### Incidence of ARDS according to Berlin Criteria and patient characteristics

The incidence of ARDS within the first 72 h of admission according to the Berlin Criteria in the present cohort was 8% (*n* = 61). The median age was 58 (48–65) years and 64% (*n* = 39) of patients were male. All patients presented with two sided pulmonary infiltrates in the chest x-ray or CT-scan. A pneumothorax was present in 5% (*n* = 3) of patients. Pulmonary congestion was observed in 39% (*n* = 24) of patients with ARDS. Pleural effusions were detected in 49% (*n* = 30) of critically ill patients with ARDS. Overall, 10% (*n* = 6) suffered from mild, 64% (*n* = 39) from moderate and 26% (*n* = 16) severe ARDS according to the Berlin Criteria. One patient received veno-venous extracorporeal membrane oxygenation (vv-ECMO) treatment due to severe respiratory failure. Risk of mortality stratified according the different ARDS severity stages is illustrated in Supp. Figure 3.

### Clinical characteristics of mechanically ventilated patients with and without ARDS

Of the 421 patients with mechanical ventilation, 14% (*n* = 61) presented with ARDS and 86% (*n* = 360) did not fulfill criteria for ARDS within the first 72 h after admission. For detailed patient characteristics see Table 3. Demographic characteristics including age and gender were distributed equally between both groups. Most common cause of admission was infection/sepsis in 41% (*n* = 25) and 14% (*n* = 51) in patients with and without ARDS, respectively. The median MELD (28 vs. 21 points, *p* = 0.006) and Child-Pugh score (11 vs. 10 points, *p* = 0.004) were significantly higher in patients with ARDS. Further liver and ICU specific scoring systems were numerically but not significantly higher in the ARDS group. 59% (*n* = 36) patients with ARDS received RRT compared with 49% (*n* = 176). ACLF on admission was present in 94% (*n* = 57) of patients with ARDS and in 96% (*n* = 345) patients without ARDS. Detailed characteristics on vital signs, laboratory characteristics and blood gas are shown in Table 3.

**Table 3:** Characteristics of the study population according presence of ARDS within patients with mechanical ventilation.

Variables	ARDS (*n* = 61)	No ARDS (*n* = 360)	*p* value
Age, years (*median; IQR)*	58 (48–65)	59 (51–65)	0.42
Sex, male (*n*,* %)*	39 (64)	226 (63)	0.86
Cause of ICU admission, (*n*,* %)*			< 0.001
- Infection/Sepsis	25 (41)	51 (14)	
- Bleeding	5 (8)	100 (28)	
- Renal failure/Hepatorenal Syndrome	2 (3)	23 (6)	
- Cardiovascular	0 (0)	3 (1)	
- Hepatic encephalopathy	6 (10)	30 (8)	
- Surgery/Intervention	8 (13)	74 (21)	
- Other	15 (25)	79 (22)	
MELD score, points (*median; IQR)*	28 (16–36)	21 (14–29)	0.006
Child-Pugh score, points (*median; IQR)*	11 (9–13)	10 (8–12)	0.004
SOFA, points (*median; IQR)*	14 (10–18)	13 (11–16)	0.32
SAPS II, points (*median; IQR)*	54 (38–69)	52 (38–64)	0.36
CLIF-SOFA, points (*median; IQR)*	15 (11–19)	14 (12–17)	0.18
CLIF-C ACLF, points (*median; IQR)*	59 (50–63)	57 (52–61)	0.19
CLIF-C ACLF _Lactate_, points (*median; IQR)*	60 (54–69)	57 (51–66)	0.07
Therapy, (*n*,* %)*			
- Vasopressor support	48 (79)	313 (86)	0.09
- Mechanical Ventilation	61 (100)	360 (100)	-
- Renal Replacement therapy	36 (59)	176 (49)	0.143
ACLF grade - on admission*, (*n*,* %)*			0.33
- No ACLF	4 (6)	12 (3)	
- ACLF grade 1	5 (8)	20 (6)	
- ACLF grade 2	12 (20)	54 (15)	
- ACLF grade 3	40 (66)	271 (75)	
Vital signs – on admission, (*median; IQR)*			
- Heart rate, bpm	89 (80–109)	86 (70–102)	0.02
- Mean arterial pressure, mmHg	75 (62–86)	74 (64–86)	0.73
- Body temperature, C°	35.9 (35.2–36.7)	35.7 (34.6–36.5)	0.12
Blood gas analysis – on admission, (*median; IQR)*			
- paO_2_, mmHg	79 (69–105)	101 (81–147)	< 0.001
- paCO_2_, mmHg	42 (33–49)	37 (29–43)	0.006
- pH, level	7.30 (7.20–7.40)	7.34 (7.25–7.41)	0.06
- Base-Excess, mmol/l	-6 (-12–0)	-5 (-11 – -1)	0.75
- Bicarbonate, mmol/l	19 (15–24)	20 (16–23)	0.46
- Lactate, mmol/l	3.1 (1.9–6.5)	2.6 (1.4–6.1)	0.21
Laboratory – on admission, (*median; IQR)*			
- Leukocytes, G/l	12.8 (9.4–19.1)	9.3 (6.2–14.7)	< 0.001
- Creatinine, mg/dl	1.6 (0.9–2.9)	1.6 (1.0–2.6)	0.99
- Bilirubin, mg/dl	5.4 (2.2–10.1)	2.4 (1.2–6.4)	< 0.001
- CRP, mg/dl	44 (24–89)	22 (8–54)	< 0.001
- INR	1.7 (1.3–2.3)	1.5 (1.2–1.9)	0.02
Outcome, (*n*,* %)*			
- LOS – ICU, days (*median; IQR)*	6 (2–13)	7 (3–16)	0.35
- LOS – Hospital, days (*median; IQR)*	14 (7–24)	19 (10–35)	0.04
- 28-day Mortality/tx (*n*,* %)*	40 (66)	196 (54)	0.11
- 90-day Mortality/tx (*n*,* %)*	43 (70)	226 (63)	0.25

*Abbreviations*: IQR, interquartile range; n, number; MV, mechanical ventilation; ICU, intensive care unit; MELD, Model of End Stage Liver Disease; SOFA, sequential organ failure assessment; SAPS, simplified acute physiology score; CLIF, Chronic Liver Failure; ACLF, Acute-on-chronic liver failure; CRP, C Reactive Protein; INR, international normalized ratio; LOS, length of stay; tx, transplantation;

* 3 missing;

### Outcome of patients with MV and ARDS

**Fig. 2:**
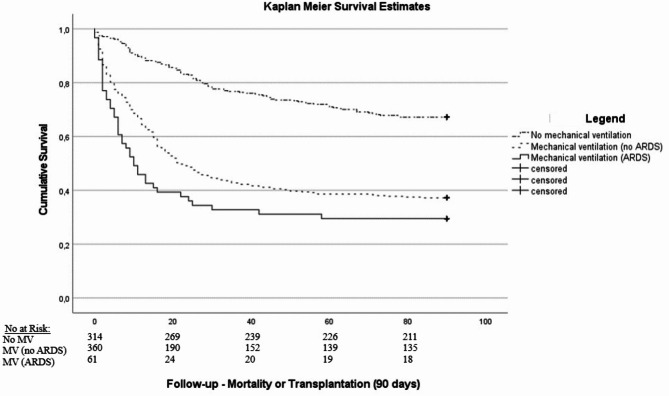
Kaplan-Meier Survival estimates stratified according no-mechanical ventilation, mechanical ventilation and ARDS (log-rank: *p* < 0.001).

We observed a 28-day mortality or liver transplantation rate of 54% (*n* = 196) and 66% (*n* = 40) in patients with MV and ARDS, respectively (see also Kaplan-Meier survival estimates Supp. Figure 2). After 90-days 63% (*n* = 226) with MV and 70% (*n* = 43) with ARDS were dead or received liver transplantation (see also Kaplan-Meier survival estimates Fig. 2 and Supp. Figure 1). The median length of hospital and ICU stay in patients with MV and ARDS was 19 (10–35) and 7 (3–16) days compared with 14 (7–24) days and 6 (2–13) days, respectively. For detailed characteristics of patients with and without ARDS are shown in Table 3.

### Factors associated with mortality in patients with mechanical ventilation

Cox regression analysis identified SAPS II (HR 1.02, 95% CI 1.008–1.028, *p* < 0.001), MELD (HR 1.07, 95% CI 1.049–1.086, *p* < 0.001), severity of ACLF (HR 0.80, 95% CI 0.646–0.991, *p* < 0.05), and requirement of RRT (HR 1.58, 95% CI 1.113–2.232, *p* = 0.01) as factors significantly and independently associated with mortality in patients with mechanical ventilation.

## Discussion

In this large cohort of critically ill patients with liver cirrhosis we found that almost 60% of patients required mechanical ventilation. Of those with mechanical ventilation 14% developed ARDS within the first 72 h after ICU admission. Presence of ARDS was associated with an increased mortality; mortality was highest among patients with stage of severe ARDS. We could identify relevant clinical characteristics and differences of mechanical ventilated patients with and without ARDS.

The mortality of critically ill patients with liver cirrhosis admitted to the intensive care unit is high^6,7^. If patients with liver cirrhosis need invasive mechanical ventilation the mortality is even worse^11^. Respiratory failure is a less frequent type of organ failure in patients with liver cirrhosis^1^. There are different factors contributing to the worsening of respiratory function in patients with liver cirrhosis which including pulmonary vascular changes (e.g. hepatopulmonary syndrome)^2–4^ or extrapulmonary changes like ascites or hepatic hydrothorax^5^. In the present study we observed, that 57% of patients required mechanical ventilation. This rate is in line with previous studies^11^. Of interest an earlier study showed that 66% of patients with mechanical ventilation did not survive the ICU stay and more importantly these patients in particular had an 1-year mortality rate of 89%^11^. Especially, when medical resources (e.g. critical care capacity) are a limited resource admissions to the ICU must be well considered. Especially, admission of patients with liver cirrhosis to the ICU without the perspective of transplantation is often considered questionable^34,35^. In the setting of respiratory failure, the development of ACLF is likely^11,36^. The development of ACLF is highly dynamic and associated with high mortality. In the current study we observed that patients with need for mechanical ventilation had a mortality of 54% after 28-days and 63% after 90-days. Compared to previous studies this is significantly lower and maybe explained by ventilation strategies as well as probably a different patient selection.

14% of patients with ACLF and mechanical ventilation developed ARDS within the first 72 h after ICU admission using the Berlin criteria. In this cohort, presence of ARDS of any stage was associated with a significantly increased short-term mortality. We observed a mortality of 66% after 28-days and 70% after 90-days. Compared to patients with mechanical ventilation without ARDS, we observed that patients with ARDS were more commonly admitted with an underlying infection. Furthermore, MELD and Child-Pugh score was higher in this collective. In an earlier study, length of ventilation was found as poor prognostic factor^11^. Although we could not find an association in our cohort this is reasonable. Furthermore, we could find a significant relationship between mechanical ventilation and ICU mortality in this cohort. This is in contrast to earlier studies and might be explained by the low sample size of the previous studies^37,11^. In the current study we showed that mortality was following severity of ARDS. Of interest a recent study investigated the outcome of ARDS in patients with and without cirrhosis and found that there was a significant increased mortality in patients with cirrhosis^12^. Furthermore, cirrhosis was independently associated with mortality, after adjustment for age, non-hepatic SOFA and PaO2/FiO2 at day one. The rate in mortality in patients with ARDS in the present study was comparable. This is of particular interest, because earlier studies proposed mortality rates reaching above 90%^38,39^.

25% of patients in the present cohort had severe ARDS. Severe ARDS is mainly characterized by the severe limitation of the gas-exchange expressed by low levels of oxygen in the blood. Selected patients, who develop progressive acute respiratory failure refractory to optimal support with conventional MV, the use of veno-venous extracorporeal membrane oxygenation (vv-ECMO) may be considered as therapy option. Early referral to ECMO centres as well as early initiation of vv-ECMO has been proven to be beneficial^40,32,41^. Thus, the use of vv-ECMO has increased substantially in critical care units during the last decade^42^. In the current cohort one patient received vv-ECMO therapy. However, one registry study investigated the use of ECMO in patients with liver cirrhosis and found an in-hospital mortality rate of 76%^43^. The data derives from a large registry and is limited because it also included patients in cardiogenic shock or after liver transplantation which makes comparisons to an underlying respiratory failure difficult. However, the study showed that the number of complications including major bleeding or need for RRT was significantly higher in patients with liver cirrhosis. Currently the role of vv-ECMO in patients with liver cirrhosis remains unclear. Treatment with vv-ECMO in patients with liver cirrhosis should not be generally withheld. However, due to a quite limited prognosis in patients with advanced stages of ACLF decision for vv-ECMO therapy should be based on different factors to ensure to select the optimal patients who will most benefit from the therapy.

This study has several limitations that should be noted. First, we show results of a center highly experienced in management of critically ill patients liver cirrhosis and ACLF. Thus, the results and conclusions may not be generally transferable to other hospitals and settings with less experience. Second, data were collected from a prospectively documented PDMS and retrospectively analyzed. Third, ARDS incidence was evaluated accordingly to daily worst paO_2_/FiO_2_ value. Thus, the incidence of ARDS may be overestimated as daily lowest paO_2_/FiO_2_ could be the result of temporary episodes of hypoxemia. Fourth, in the present study we did not investigate the etiology of ARDS which can be multifactorial in patients with liver cirrhosis including pneumonia, aspiration and other lung-liver interactions. Fifth, the cause of intubation was not documented but followed local and international protocols. The results may not be generally transferable to other hospitals and settings. Sixth, due to the timespan covered in this study also management practices of MV and ARDS have changed which could have had an impact on outcome of the included patients. Seventh, residual confounding from unmeasured covariables is a matter of concern and cannot be entirely excluded.

## Conclusions

We could show that ARDS is a poor prognostic factor for patients with liver cirrhosis admitted to the ICU. Furthermore, about 10% of critically ill patient with cirrhosis admitted at the ICU develop ARDS within the first 72 h after ICU admission. The development of ARDS contributes to significantly increased 28-day mortality. Although mortality rates are high therapy should not be withheld and must be reevaluated regularly.

## Electronic supplementary material

Below is the link to the electronic supplementary material.

Supplementary Material 1

Supplementary Material 2

Supplementary Material 3

Supplementary Material 4

## Data Availability

The datasets supporting the conclusions of this article are included within the article.
