# Supplementary figures and images for: Impact of acute respiratory distress syndrome on outcome in critically ill patients with liver cirrhosis
Source: Sci Rep. 2025 Feb 4;15:4301. doi: 10.1038/s41598-025-88606-z (PMC11794433; doi:10.1038/s41598-025-88606-z)

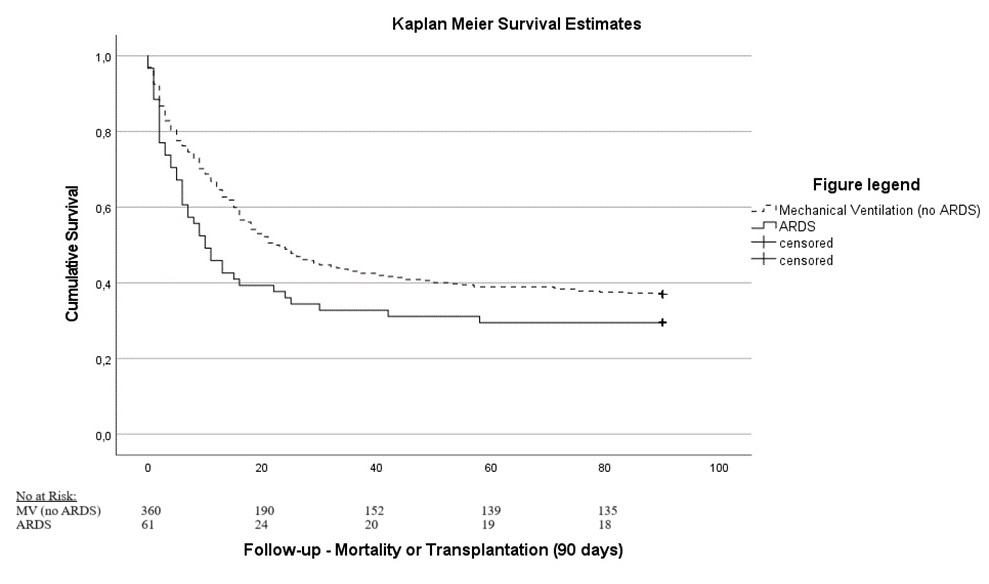

Supplement: Supplementary file 1 — Supplementary Material 1 [file 41598_2025_88606_MOESM1_ESM.jpg]

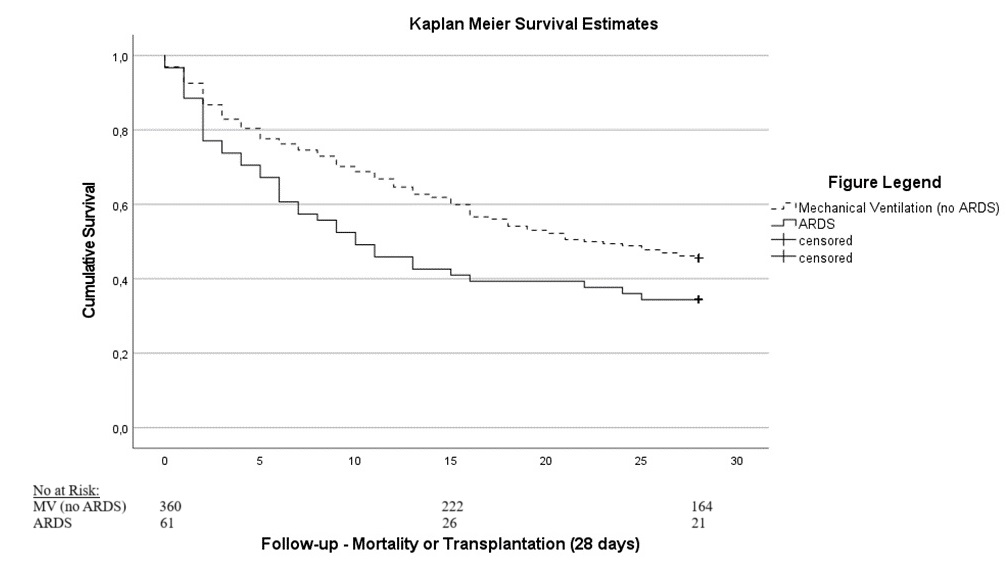

Supplement: Supplementary file 2 — Supplementary Material 2 [file 41598_2025_88606_MOESM2_ESM.jpg]

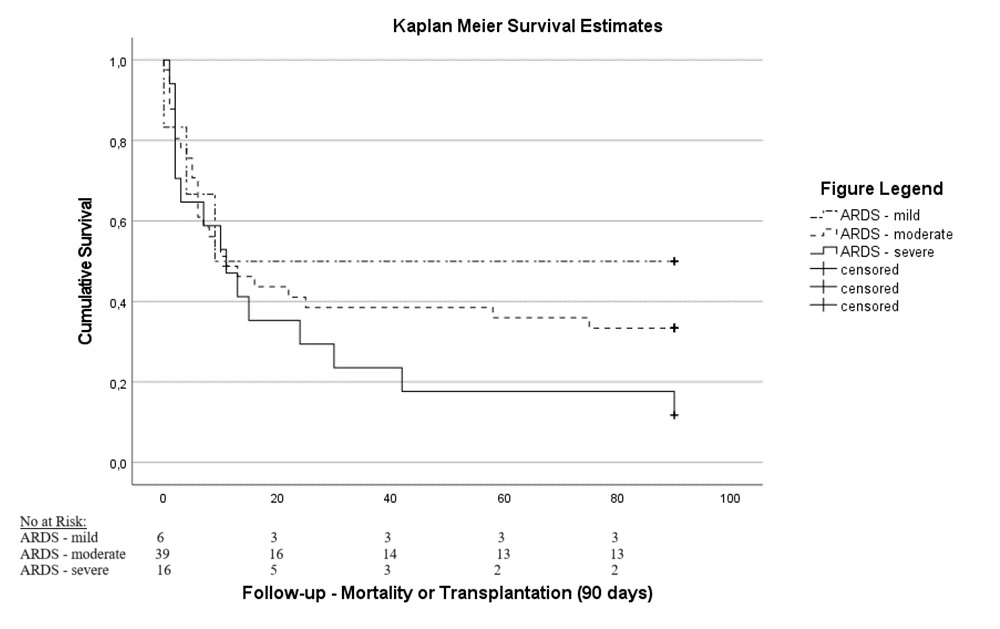

Supplement: Supplementary file 3 — Supplementary Material 3 [file 41598_2025_88606_MOESM3_ESM.jpg]
