# Supplementary material for: Impact of acute respiratory distress syndrome on outcome in critically ill patients with liver cirrhosis
Source: Sci Rep. 2025 Feb 4;15:4301. doi: 10.1038/s41598-025-88606-z (PMC11794433; doi:10.1038/s41598-025-88606-z)
Supplement: Supplementary file 4 — Supplementary Material 4 [file 41598_2025_88606_MOESM4_ESM.docx]

**Supplementary - Figure Legends**

**Supp. Fig. 1:** Kaplan-Meier Survival estimates for patients with mechanical ventilation; stratified according mechanical ventilation and ARDS (log-rank: p = 0.052)

**Supp. Fig. 2:** Kaplan-Meier Survival estimates for patients with mechanical ventilation; stratified according mechanical ventilation and ARDS (log-rank: p = 0.024)

**Supp. Fig. 3:** Kaplan-Meier Survival estimates for patients with ARDS; stratified according ARDS severity stages (log-rank: p = 0.369)
